# Analysis of traumatic injuries presenting to a referral hospital emergency department in Moshi, Tanzania

**DOI:** 10.1186/1865-1380-5-28

**Published:** 2012-06-08

**Authors:** Erica R Casey, Florida Muro, Nathan M Thielman, Elifuraha Maya, Eric W Ossmann, Michael B Hocker, Charles J Gerardo

**Affiliations:** 1 Division of Emergency Medicine, Duke University School of Medicine, Durham, NC, USA; 2 Hubert Yeargan Center for Global Health, Duke University, Durham, NC, USA; 3 Duke Global Health Institute, Duke University, Durham, NC, USA; 4 Kilimanjaro Christian Medical Centre, Kilimanjaro Christian Medical College, Moshi Tanzania, East Africa

**Keywords:** World health, Injuries, Emergency medicine, Motor vehicles, Accidental falls

## Abstract

**Background:**

Injuries represent a significant and growing public health concern in the developing world, yet their impact on patients and the emergency health-care system in the countries of East Africa has received limited attention. This study evaluates the magnitude and scope of injury related disorders in the population presenting to a referral hospital emergency department in northern Tanzania.

**Methods:**

A retrospective chart review of patients presenting to the emergency department at Kilimanjaro Christian Medical Centre was performed. A standardized data collection form was used for data abstraction from the emergency department logbook and the complete medical record for all injured patients. Patient demographics, mechanism of injury, location, type and outcomes were recorded.

**Results:**

Ten thousand six hundred twenty-two patients presented to the emergency department for evaluation and treatment during the 7-month study period. One thousand two hundred twenty-four patients (11.5%) had injuries. Males and individuals aged 15 to 44 years were most frequently injured, representing 73.4% and 57.8%, respectively. Road traffic injuries were the most common mechanism of injury, representing 43.9% of injuries. Head injuries (36.5%) and extremity injuries (59.5%) were the most common location of injury. The majority of injured patients, 59.3%, were admitted from the emergency department to the hospital wards, and 5.6%, required admission to an intensive care unit. Death occurred in 5.4% of injured patients.

**Conclusions:**

These data give a detailed and more robust picture of the patient demographics, mechanisms of injury, types of injury and patient outcomes from similar resource-limited settings.

## Background

Traumatic injuries represent a significant and growing disease burden in the developing world, and now represent one of the leading causes of death in economically active adults in many low- and middle-income countries. Five of the top 15 causes of mortality in adults aged 15 to 29 years now result from injuries. In low-income countries long-term morbidity from injuries, as defined by disability-associated life years (DALY), is higher than morbidity caused by either cardiovascular conditions or malignancies [1]. This trend will continue as road traffic injuries (RTI) alone are projected to be the sixth leading cause of death and third highest cause of DALY by 2020 [2].

Likewise, the economic impact of injuries in low-income countries for individuals and for society as a whole is disproportionately high. The total economic costs amount to as much as 65 billion US dollars, more money than low-income countries receive in total assistance aid annually [3]. The cost-effectiveness of injury prevention and emergency treatment of injury in these resource-limited settings is not yet well understood as the development of emergency care systems is in its nascency.

Despite the disproportionate numbers of death and disabilities caused by injuries in low- and middle-income countries, the burden of disease resulting from these events has been largely under-reported in the emergency medicine literature [4,5]. Moreover, the lack of consistent reporting of injuries leads to significant underestimates of the morbidity and mortality in these settings [6]. Poor mechanisms for reporting combined with inability to afford medical evaluation after an injury are likely important contributors to this phenomenon. Few studies have been performed in low-income countries, in particular in sub-Saharan Africa, to characterize the mechanisms of injury as well as the types of injury sustained in these events. Early data on the epidemiology of injury in East Africa, specifically in Tanzania, are just emerging, and the full scope of the problem is not yet well understood [7].

Effective injury prevention, patient care and rehabilitation all require a prior understanding of injury epidemiology in order to develop an effective response. Establishing hospital-based data collection systems, as recommended in the World Health Organization (WHO) injury surveillance guidelines, may contribute to a fuller understanding of injuries in low-income countries [8,9]. Additionally, there has been a recommendation for emergency care research, including hospital chart review, to begin to address the paucity of epidemiologic injury data in these settings [4]. This study evaluates the magnitude and scope of injury-related disorders in the population presenting to a large consultant and referral hospital emergency department in northern Tanzania.

## Methods

### Study design

We performed a retrospective chart review of patients presenting with traumatic injuries to the Kilimanjaro Christian Medical Centre (KCMC) Casualty Department using established recommendations for data collection from medical records [10]. This study received Duke Institutional Review Board exemption from full review, KCMC Ethics Committee approval and exemption from review by the Tanzania National Institute of Medical Research.

### Setting

Tanzania is situated in the East African region, bordered in the north by Kenya, in the west by Uganda, the Democratic Republic of Congo, Rwanda and Burundi, and to the south, by Zambia, Malawi and Mozambique. Currently 4 consultant specialty referral hospitals and 17 regional hospitals on the mainland serve a population of over 37 million people. Emergency departments in this region are typically referred to as casualty departments and are staffed by physicians, referred to as registrars, who have completed 5 years of medical school and have undergone a 1-year internship program. Until 2010, when the first emergency medicine residency-training program was initiated at Muhimbili Hospital in Dar es Salaam, no formal emergency medicine physician training was available in Tanzania.

KCMC, located in Moshi, Tanzania, is the third largest hospital in the country, with 500 inpatient beds, and serves as the referral hospital for northeastern Tanzania. The KCMC Emergency Department has six beds and one large resuscitation room containing two additional beds. Over 17,000 patients are evaluated annually in this setting. The facility is staffed by four nurses per shift. Three registrar physicians provide care during the day, and an intern covers the department overnight. During the period of this study the physicians staffing the emergency department had no specialty emergency medicine or critical care training.

There is no established prehospital system in the region. There are ambulances available for inter-facility transfer to this large referral hospital. However, this is reserved for the most severely injured patients. The majority of patients arrive via public transportation, private auto, motorcycle or taxi, and infrequently by police.

### Selection of patient records

The emergency department maintains a logbook of every patient presenting for care. All patients presenting to the department with a chief complaint of injury, as defined by the International Classification of Disease 9th revision (ICD-9): External Causes of Injury E800-E999, were subject to chart review. Patient records initially identified in the emergency department logbook were excluded from the subsequent analysis if the complete medical record could not be located for review.

### Data collection

Data abstraction of the complete medical record was performed using a standardized data collection form, based on recommendations by the WHO injury surveillance guidelines (Additional file 1) [8]. A second independent abstractor reviewed a convenience sample of 15 percent of the patient charts in order to assess the inter-rater reliability of our data collection tool.

Patient demographics were obtained from the emergency department logbook and verified against the complete medical record to ensure that the appropriate chart was reviewed. Mechanism of injury, location of injuries by organ system, type of injuries, disposition and outcomes of patients were then documented after review of the emergency department log book and the complete medical record. Only injuries related to the initial trauma were recorded in the data collection forms, while complications secondary to hospitalization or return visits were not included for analysis. If an injury was suspected based on the emergency department logbook documentation, but not definitively identified in the medical record, then it was recorded as no injury. If multi-system injury was sustained all locations and types of injury were recorded. Classification of injury type was based on review of complete patient medical records, including history, physical exam, definitive diagnoses, radiologic studies, intra-operative examination, and pathological examination.

### Outcome measures

Mortality was defined as an injury-related death prior to arrival to the hospital, death prior to hospital admission from the emergency department or death during hospitalization. The severity of injury was determined using the WHO Injury Surveillance Guideline coding recommendations: (1) no apparent injury; (2) minor or superficial injury; (3) moderate, requiring some skilled treatment (e.g., sutures); (4) severe, requiring intensive medical/surgical management [8]. The definition of a surgical intervention included fracture reductions, laceration repairs and all procedures requiring intervention in the operating theater. Duration of hospitalization was measured in days and defined as time spent in the hospital, from evaluation in the casualty department to the day of discharge from the hospital or expiration day. Patients observed in the hospital for less than 24 h or who expired in less than 24 h were considered to have a 1-day length of hospitalization. Patients who were evaluated in the casualty department and then discharged to home were considered to have 0 days of hospitalization. Disposition from the emergency department was categorized as treated and released, admission to the hospital, transfer to another facility or death in the emergency department.

### Statistical analysis

Descriptive data from the 998 available complete patient records were analyzed using the R Environment for Statistical Computing and Graphics [11]. Means and standard deviations (SD) were used to describe continuous variables. Frequencies and proportions were used to describe categorical variables with confidence intervals of the proportions determined. Inter-rater reliability of the data collection tool was evaluated using Cohen’s kappa coefficient. Correlates of mortality and severity of illness were reported as crude odds ratios and calculated using logistic regression in JMP® Pro 9.0.

## Results

**Figure 1:**
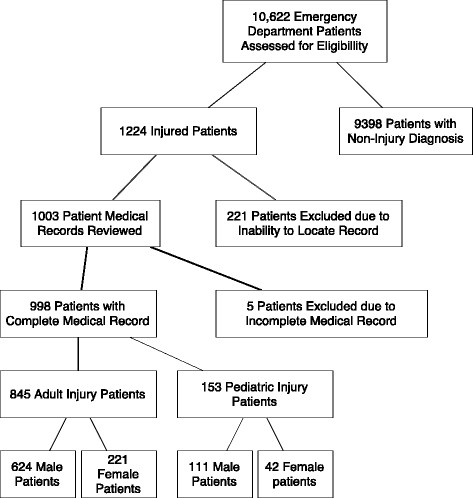
Patient medical record selection.

During a 7-month time period, dating 1 January 2010 to 31 July 2010, 10,622 patients presented to the emergency department at KCMC for evaluation and treatment. One thousand two hundred twenty-four patients were identified from the emergency department logbook with an injury-related diagnosis, representing 11.5% of all visits. Of the 1,224 patients initially identified, 1,003 medical charts were located. Two hundred twenty-six patients were not included because of missing medical records or incorrectly documented medical record numbers. Nine hundred ninety-eight medical records were adequately complete for data analysis (Figure 1). Based on the emergency department logbook, the patients with missing medical records were slightly older, with an average age of 40.1 years. Otherwise, they were similar, with 72.7% males, 26.2% and 34.6% with head injury and extremity injury, respectively, and 57% requiring hospitalization. Kappa coefficients of data points of the collection tool ranged from 0.985 to 1, indicating excellent agreement between the two reviewers.

### Patient demographics

**Figure 2:**
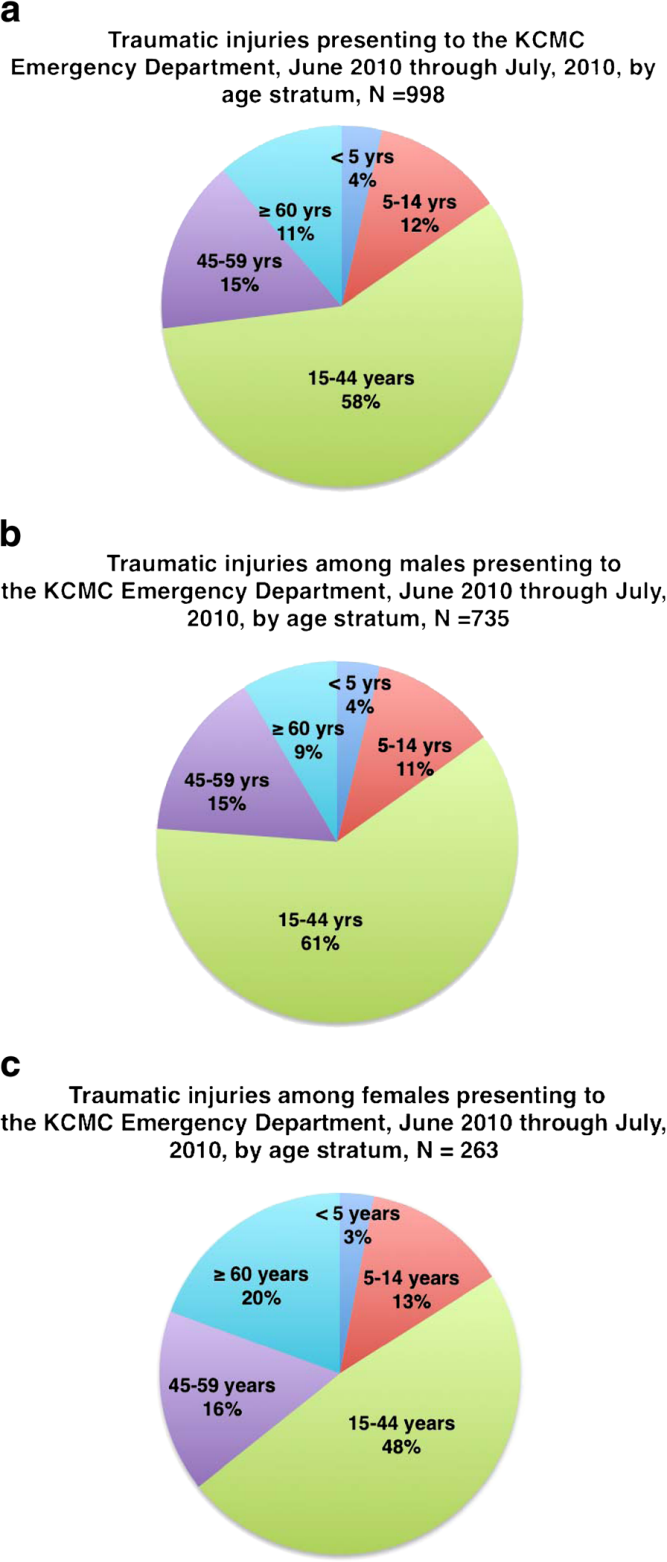
**a. Traumatic injuries presenting to the KCMC Emergency Department, June 2010 through July, 2010 by age stratum,*N*** **= 998. b.** Traumatic injuries among males presenting to the KCMC Emergency Department, June 2010 through July, 2010, by age stratum, *N* = 735. **c.** Traumatic injuries among females presenting to the KCMC Emergency Department, June 2010 through July, 2010 by age stratum, *N* = 263.

The mean age of injured patients in the population was 34.6 (SD 19.9) years. The average injured patient age was younger if male, at 33.1 (SD 18.3) years, compared to females whose average age was 39.1 (SD 23.4) years. Males and individuals aged 15 to 44 years represented the largest number of injuries presenting to the hospital for evaluation (Figure 2a-c). The majority, 59%, of patients were referred from hospitals, clinics, and dispensaries in the Kilimanjaro region, for a variety of reasons, including primary surgical or orthopedic care, worsening condition or complications occurring during hospitalization at another facility. Sixty-six different health facilities were documented as the transfer facility to this referral hospital, with nearly half, 42.8%, referred from the closest district hospital. The majority of referrals arrived via private vehicle or public transportation. Inter-facility transfer to the KCMC Casualty Department is not uncommon.

### Mechanisms of injury

**Figure 3:**
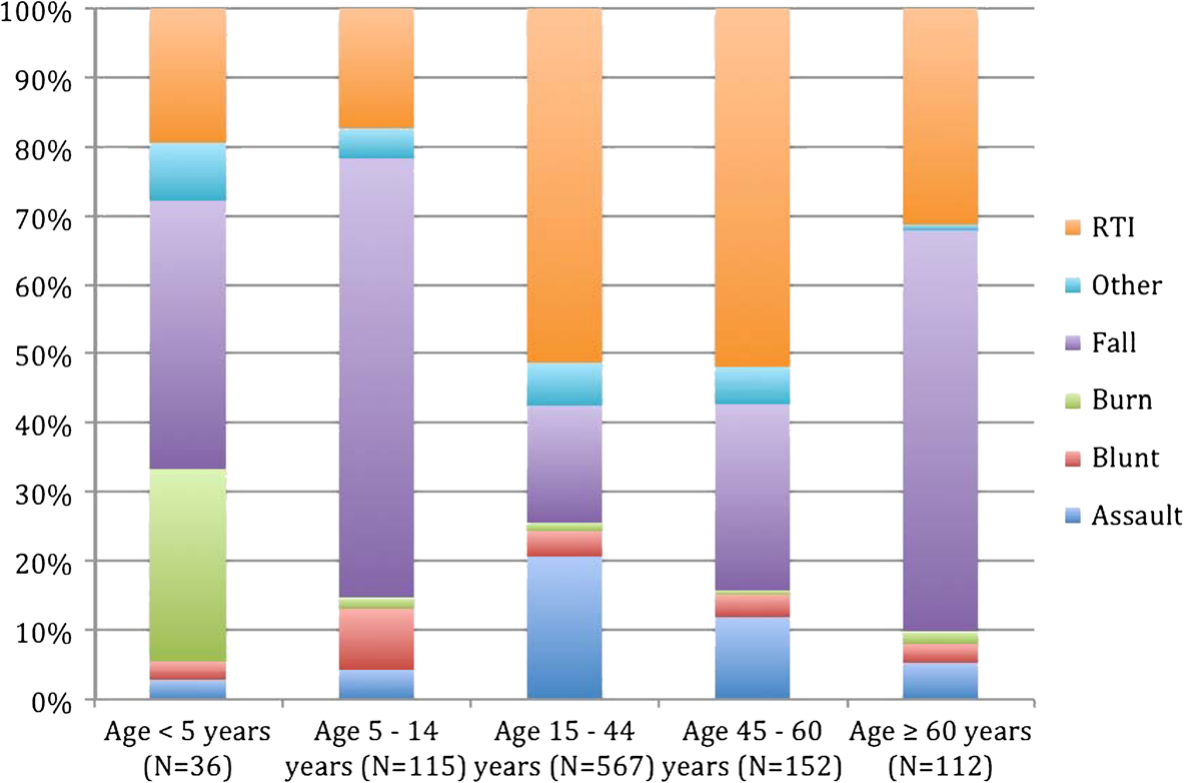
Mechanism of traumatic injuries within age stratum, proportion.

Road traffic injuries (RTI) represent the largest proportion of traumatic events in the patients presenting to the emergency department for medical care. Four hundred thirty-two patients, 43.9% (95% CI 40.9, 47.1), reported involvement in an RTI as the antecedent mechanism of injury. Categorization of RTI by road traffic user suggests that motorcyclists and pedestrians were the two most common populations impacted by these events. However, over one-third of the records, 38.7%, of the road traffic-related injury subjects did not have adequate documentation in the medical record to determine the precise category of RTI. Injuries related to a fall and secondary to an assault were the second and third most common mechanisms reported at 29.5% (95% CI 26.7, 32.4) and 15.0% (95% CI 12.9, 17.3), respectively (Figure 3).

Patients aged 15 to 44 years represented the highest proportion of injuries for all mechanisms, except falls and burns. Falls were more common in older patients over 45 years of age, representing 36.7% (95% CI 31.2, 42.6) of fall-related injuries. Burns were most common in children under 5 years of age, 45.5% (95% CI 25.2, 67.3). There was a distinctively male majority for nearly all mechanisms of injury except bite wounds and poisoning.

### Location and type of injury

Evaluation of the injury location demonstrates a significant pattern of head/neck or extremity injury as either isolated or multi-system injury. Head injuries accounted for 21.0% (95% CI 18.6, 23.7) of isolated injuries and were involved in 15.0% (95% CI 12.9, 17.45) of patients suffering multi-organ system injury. Likewise, injured extremities accounted for almost half, 46.4% (95% CI 43.3, 49.5), of all isolated traumatic injuries and 13.1% (95% CI 11.1, 15.4) of multi-organ system injuries.

Consistent with injury location, the most common types of injury were fractures and traumatic brain injury (TBI). Fractures of the extremities occurred in 44.1% (95% CI 41.0, 47.2) of injured patients, most of which, 77.2%, were closed. TBI, including patients with intracranial hemorrhage and skull fractures, were documented in 28.7% (95% CI 25.9, 31.6) of injured patients. Blunt trauma resulted in more injuries to the chest, abdomen and pelvis including lung contusions, hemo-pneumothoraces, solid organ injuries and visceral injuries. The majority of injuries, 81%, were documented as unintentional trauma.

### Injury severity

Head injury was associated with increased mortality, OR 5.76 (95% CI 2.57, 12.92). No other injury location was clearly associated with mortality (Table 1). Head, abdominal and spinal injury were also associated with severe injury, OR 3.31 (95% CI 2.23, 4.98), 6.04 (95% CI 3.45, 10.44) and 19.95 (95% CI 6.55, 73.75), respectively, as defined by the WHO Injury Surveillance Guidelines.^8^ No specific mechanism of injury was clearly associated with higher mortality or severity of injury.

**Table 1:** Mortality by Injury Location

**Location**	*N*	Proportion^*^ (95% CI)	**Male: female ratio**	**Number of deaths**	**Crude OR for mortality**
**Head and neck**	353	36.5 (33.6, 39.6)	3.3	25	5.76 (2.57, 12.92)
**Chest**	125	12.9 (11.0. 15.2)	2.5	6	1.52 (0.61, 3.76)
**Back**	24	2.5 (1.7, 3.7)	2.4	1	1.24 (0.16, 9.44)
**Abdomen and pelvis**	62	6.4 (5.0, 8.1)	4.1	3	1.48 (0.44, 5.00)
**Extremity**	575	59.5 (56.4, 62.6)	2.5	13	0.43 (0.21, 0.87)
**Spine**	14	1.4 (0.9, 2.4)	6.0	0	--

*Total > 100% as some patients with multiple injuries

### Patient disposition and outcomes

The majority of injured patients, 59.3% (95% CI 56.1, 62.3), were admitted from the emergency department at to the hospital wards. A minority of patients, 5.6% (95% CI 4.4, 7.4), required admission to an intensive care unit. The remainder were evaluated in the emergency department, and subsequently treated and released without hospitalization. During hospitalization a substantial minority, 37.8% (95% CI 33.9, 40.0), of patients required surgical intervention. Death, either prior to arrival in the emergency department, while in the emergency department or during hospitalization, occurred in 5.4% (4.2, 7.0%) of patients suffering from injuries. The average length of hospital stay was 9.5 days with a 3-day median length of stay.

## Discussion

Injury prevention and improved quality of emergency care for injured patients necessitates an understanding of the attributable mechanisms of injury and their resulting impact on patients, families and the broader health care system on which they depend. As injuries have become more significant causes of morbidity and mortality in the developing world, few mechanisms for improvement in data collection, primary prevention, or improved pre-hospital and emergency care have emerged [12]. Our review of injuries in patients presenting to KCMC establishes a significant pattern of injury in the population and is consistent with injury patterns reported in other low-income countries [7,12-14]. In our study, RTI was the major mechanism of injuries in patients presenting to the hospital, accounting for 43.2% of all injuries. This was within the range of prior studies in Uganda, Ghana and Tanzania reported as 49%, 29% and 33%, respectively. Furthermore, the burden of RTI-related injury morbidity and mortality is inequitably distributed globally [15-21]. While low- and middle-income countries comprised only 32% of the world’s vehicle ownership in 2000, they accounted for more than 85% of the global burden of motor vehicle fatalities and 90% of DALY [21]. This is likely related to increased motorization in the setting of inadequate transportation infrastructure maintenance, lack of safety device use such as restraints and helmets, mixed road use by pedestrians or non-motorized vehicles, and the absence of pre-hospital and emergency care.

Furthermore, this study suggests that motorcyclists are involved in a substantial number of RTIs. Previous studies have suggested that pedestrians and public transport users are the groups most significantly impacted by RTI; however, these data suggest a potential increasing burden of motorcycle-related RTI as their use increases. This has significant implications for the potential impact of specific interventions as injury prevention efforts focusing on enforcement of helmet usage, speed limit enforcement and provision of safe footpaths alongside roads are potentially important mechanisms for reduction of injury numbers [21]. Currently, Tanzania does have a national helmet law for motorcyclists; however, usage is not consistently enforced and does not apply to all vehicle occupants.

Falls and assaults were the second and third most common cause of injuries, and are also likely high-yield areas for intervention. Although burns accounted for a small percentage of patient injuries, they are of particular concern in children less than 5 years of age, who suffered 45.5% of all burns. This likewise represents another potential focus for prevention.

The types of injuries, which predominantly involve trauma to the head and extremities, underscore the need for prevention measures as well as potentially improving availability of medical services. Limited access to qualified specialists results in significant treatment delay and potentially higher morbidity and mortality, however the impact of improving pre-hospital or hospital level care services is still uncertain when compared to prevention mechanisms such as improved road safety regulations. One study in Trinidad where trauma life support courses (ATLS) were instituted for physicians and pre-hospital care providers indicated a significant reduction in mortality from injuries, suggesting educational programs directed towards existing medical care providers may be an effective, affordable mechanism for reducing morbidity and mortality from injuries [22].

Finally, our results demonstrate the significant impact injuries have in males between the second and fourth decade of life. In our study, men accounted for 72.7% of the patients presenting to the emergency department with injuries and 57.8% of patients were between the ages of 15 and 45 years. As injuries often result in significant morbidity with potential for long-term disability and inability to return to prior work activities, the impact on the individual and their family’s socioeconomic status can be substantial [23,24]. Pre-injury socio-economic status of the patient has been suggested as a determinant of injury risk in developing countries previously. Future prospective studies in the Kilimanjaro region should include measures of the economic impact and burden of injury in the country and underscore the need for measures focusing on injury prevention and emergency care.

This study has several limitations, as it is a retrospective chart review of the medical records which may contain missing, incomplete, or conflicting data. Moreover, there is inherent risk of either unreliable or biased data abstraction with this methodology. However, we minimized these effects by using strict definitions and processes for handling data points as previously described and assessed reliability of our data abstraction tool. The data abstractors were not blinded to the goals of our investigation.

Additionally, KCMC is a referral hospital for the Kilimanjaro, Tanga, Arusha, and Manyara regions and thus often receives referrals or transfer patients from dispensaries, clinics, and hospitals throughout the region. This may contribute to a selection bias and limits the generalizability of these hospital-based epidemiology results to the overall population or other healthcare sites. This affect would tend to skew the data towards more severely injured patients, which is supported by the low number of lacerations, bites, and other minor injuries. Conversely, limited access to pre-hospital care services or limited transportation from more remote sites potentially negatively impacts delivery of patients to health care centers with severe or morbid injuries. As such, many injury-related deaths may be unaccounted for in hospital records resulting in an underestimation of the true morbidity and mortality related to injury.

## Conclusions

Our study demonstrates that a substantial minority of patients presenting to a Tanzanian referral hospital’s emergency department have sustained injury. These data give a detailed and more robust picture of the patient demographics, mechanisms of injury, types of injury, and patient outcomes from similar resource limited settings. Additionally they demonstrate the need for prospective data collection, injury prevention, and development of emergency medical care strategies in low-income countries.

## Abbreviation

DALY, Disability Associated Life Years; KCMC, Kilimanjaro Christian Medical Centre; RTI, Road Traffic Injury; TBI, Traumatic Brain Injury; WHO, World Health Organization.

## Competing interests

The authors declare that there are no competing interests.

## Authors’ contributions

EJC, NMT and CJG conceived and drafted the initial study design. ERC, CJG, NMT < FM, EWO and MBH revised the study design. ERC, FM and EM, supervised the conduct of the study and data collection. ERC and NMT analyzed the data. ERC drafted the manuscript and all authors contributed substantively to its revision. ERC takes responsibility for the manuscript as a whole. All authors read and approved the final manuscript.

## Supplementary Material

Additional file 1 Data Collection Form. Click here for file
