# Supplementary material for: Analysis of traumatic injuries presenting to a referral hospital emergency department in Moshi, Tanzania
Source: Int J Emerg Med. 2012 Jun 8;5:28. doi: 10.1186/1865-1380-5-28 (PMC3407759; doi:10.1186/1865-1380-5-28)
Supplement: Additional file 1 — Data Collection Form. [file 1865-1380-5-28-S1.pdf]

Additional File 1: Data Collection Form

Study ID Number:

Sex: Male ☐ Female ☐

Age (Date of birth):

Occupation:

Place where injury occurred:

Time of injury (Days prior to hospital arrival:

Patient arrival time to medical facility:

Mechanism of injury:
Road Traffic injury:
- Private vehicle ☐
- Public vehicle ☐
- Motorcyclist ☐
- Bicyclist ☐
- Pedestrian☐

Gunshot / Stabbing ☐
Fall ☐
Bite☐
Burn ☐
Stabbing ☐

Injury intent:
Unintentional ☐
Intentional ☐
Unknown ☐

Location of injury: (Check all that apply)
None ☐
Head & neck ☐
Back ☐
Spine ☐
Face ☐
Chest ☐
Abdomen ☐
Pelvis ☐
Urogenital ☐
Extremity ☐

Type of injury: (Check all that apply)
None ☐
Bite ☐
Burn ☐
Cold-related(frost bite, nip, gangrene) ☐
Concussion ☐
Contusion ☐
Fracture ☐
Ingestion of toxic substance ☐
Laceration ☐
Paresis / Paralysis ☐
Solid organ injury ☐
Sprain / Strain ☐

Systolic blood pressure on arrival:

Pulse rate on arrival:

Respiratory rate on arrival:

Neurological status on arrival:
Alert, no neurologic impairment ☐
Responsive to verbal stimuli ☐
Responsive to painful stimuli ☐
Unresponsive ☐

Patient disposition:
Treated and released ☐
Death in the casualty ward ☐
Admission to the hospital ☐
Transferred to other facility ☐

Hospitalization course:
Surgical intervention ☐
Non-surgical care ☐
Death during hospitalization ☐

Duration of hospitalization: (days):

Diagnoses during hospitalization:

Surgical interventions:
